# A new species of spotted leaf frog, genus *Phasmahyla* (Amphibia, Phyllomedusidae) from Southeast Brazil

**DOI:** 10.7717/peerj.4900

**Published:** 2018-05-30

**Authors:** Elvis Almeida Pereira, Lucas Custódio Lomba Rocha, Henrique Folly, Hélio Ricardo da Silva, Diego José Santana

**Affiliations:** 1 Departamento de Biologia Animal, Universidade Federal Rural do Rio de Janeiro, Seropédica, Rio de Janeiro, Brazil; 2 Curso de Ciências Biológicas, Centro Universitário Redentor, Itaperuna, Rio de Janeiro, Brazil; 3 Departamento de Biologia Animal, Universidade Federal de Viçosa, Viçosa, Minas Gerais, Brazil; 4 Instituto de Biociências, Universidade Federal de Mato Grosso do Sul, Campo Grande, Mato Grosso do Sul, Brazil

**Keywords:** Taxonomy, Systematic, Atlantic Forest, Brazil, Conservation

## Abstract

Based on concordant differences in male advertisement call, tadpole morphology, and absence of haplotype sharing in the barcoding 16S mitochondrial DNA, we describe here a new species of spotted leaf frog of the genus *Phasmahyla* from Atlantic Forest, State of Rio de Janeiro, Southeast Brazil. The new species is most similar to *P. cochranae* (type locality) and *P. spectabilis* (type locality). It differs from these species by the size of the calcar, moderate-sized body (snout-vent length 30.4–34.4 mm in adult eight males), and in the advertisement call. The tadpoles of *Phasmahyla lisbella* sp. nov. differ from *P. exilis*, *P. spectabilis*, *P. timbo*, *P. guttata* and *P. jandaia* because they do not have row of teeth in the anterior part; differ from *P. cruzi* by the shape of the anterior end of the oral disc. Through genetic data (phylogenetic distance and haplotype genealogy) we diagnosed the new species where the genetic divergences among its congeners is about 3–6% in a fragment of the 16S rRNA gene, which is above the threshold typically characterizing distinct species of anurans. However, the new species can be distinguished from other congeneric species based on an integrative approach (molecular, bioacoustics, larval, and adult morphology).

## Introduction

The Atlantic Rain Forest of Brazil is known for its remarkable diversity and endemism of anurans, harboring a large number of endemic species, commonly in remaining small and isolated forest patches (Becker et al., 2007; Haddad et al., 2013; Ferreira, Dantas & Tonini, 2012). Despite flagged as a “biodiversity hotspot” (Myers et al., 2000), the Atlantic Forest is still been degraded at a fast pace (Ribeiro et al., 2009). In the last decades, many studies revealed a large number of amphibian species occurring in the Atlantic Forest (Giam et al., 2012), and previously unrecognized lineages (Fouquet et al., 2012; Gehara et al., 2013; Tonini, Costa & Carnaval, 2013; Menezes et al., 2016). However, this ecoregion’s diversity is presumably far from being completely described considering the high rate at which new taxa are discovered (Canedo & Pimenta, 2010; Silva & Alves-Silva, 2011; Silva & Ouvernay, 2012; Lourenço-de-Moraes et al., 2014; Pimenta et al., 2014).

The spotted leaf frogs of the genus *Phasmahyla* Cruz, 1990, is one of the several endemic genera occurring in the Atlantic Forest. These small and delicate phyllomedusines (snout to vent length 29.0–45.5 mm) are characterized mainly by their indistinct external vocal sac, cream iris, and tadpoles that are neustonic with an oral disc modified into a dorsally oriented short funnel-shaped structure (Bokermann & Sazima, 1978; Cruz, 1990; Altig & McDiarmid, 1999), presumably adapted to feed at the water surface (Cruz, 1982, 1990), in addition to considerable mitochondrial DNA variation (Faivovich et al., 2010).

The genus is currently composed by seven species: *P. cochranae* (Bokermann, 1966), *P. cruzi* (Carvalho-e-Silva, Silva & Carvalho-e-Silva, 2009), *P. exilis* (Cruz, 1980), *P. guttata* (Lutz, 1924), *P. jandaia* (Bokermann & Sazima, 1978), *P. spectabilis* (Cruz, Feio & Nascimento, 2008), and *P. timbo* (Cruz, Napoli & Fonseca, 2008). The species of *Phasmahyla* inhabit mountain streams along the Atlantic Forest from the southern of Bahia to the eastern of Paraná states, including the eastern of Minas Gerais state (Cruz, 1990; Cruz, Napoli & Fonseca, 2008; Cruz, Napoli & Fonseca, 2008; Frost, 2017). All members of the genus show highly specialized reproductive strategies by laying their eggs outside the water in nests built up with enrolled leaves (Cruz, 1990). Subsequently, hatching tadpoles drop into lotic water, where they feed until they complete their metamorphosis (reproductive mode 25 *sensu* Haddad & Prado, 2005).

During field expeditions to Área de Proteção Ambiental Ventania, in the Municipality of Miracema, Northwest State of Rio de Janeiro (Brazil), we found individuals belonging to the genus *Phasmahyla*, which were not attributed to any described species. In this paper, based on an integrative approach (molecular, bioacoustics, larval, and adult morphology), we describe this population as a new species of *Phasmahyla*.

## Materials and Methods

### Sampling

We collected specimens by visual search on September and October of 2017 at Fazenda Ventania (21°20′7.62″S, 42°12′15.40″W; *datum* WGS84; 536 m above sea level), located in the Área de Proteção Ambiental Ventania, Miracema municipality, Rio de Janeiro state, Brazil (SISBIO 54493-7). The collected frogs were killed using a liquid solution of 2% lidocaine chlorhydrate, fixed in 10% formalin, and transferred to permanent storage in 70% ethanol. We also collected tissue samples (muscle and liver) before specimen fixation and stored them in 100% ethanol. Vouchers are housed in the Coleção Zoológica da Universidade Federal de Mato Grosso do Sul (acronym ZUFMS-AMP), Campo Grande, Brazil and in the Museu de Zoologia João Moojen (acronym MZUFV) at Universidade Federal de Viçosa, Viçosa, Brazil. We also examine specimens of *Phasmahyla* from the following collections: Coleção de Anfíbios do Centro de Coleções Taxonômicas da UFMG (acronym UFMG-AMP), Coleção de Anfíbios do Museu de Biologia Mello Leitão (acronym MBML), Celio F.B. Haddad Collection, Departamento de Zoologia, Universidade Estadual Paulista, Campus de Rio Claro (acronym CFBH), Coleção de Anfíbios do Museu de Zoologia da UNICAMP (acronym ZUEC-AMP), Museu de Zoologia da Universidade Estadual de Santa Cruz, Ilhéus, BA, Brazil (acronym MZUESC), Coleção do Laboratório de Herpetologia da Universidade Federal Rural do Rio de Janeiro (acronym RU-GIR).

### Bioacoustics

We recorded the advertisement calls of three males (total of 36 calls) at the type locality. Calls were recorded with a Tascam DR-40 digital recorder. Recordings were made at 21:10 h (air temperature 21.3 °C). We digitalized the recordings at 44.1 kHz, resolution of 16 bits. We analyzed calls in Raven Pro 1.5 for Mac (Bioacoustics Research Program, 2012) with the following parameters: FFT window width = 256, Frame = 100, Overlap = 75, and flat top filter and constructed audio spectrograms in R using the package seewave (Sueur, Aubin & Simonis, 2008) with the following parameters: FFT window width = 256, Frame = 100, Overlap = 75, and flat top filter. We analyzed call duration, pulse number per call and dominant frequency. Terminology of call descriptions follows Köhler et al. (2017). Comparative data for other species were obtained from the available literature (see Cruz, Napoli & Fonseca, 2008; Dias et al., 2011).

### Morphology and measurements

The measurements cited are as follows: snout-vent length (SVL), head length (HL), head width (HW), interorbital distance (IOD), eye-nostril distance (END), eye-snout distance (ESD), internarial distance (IND), eye diameter (ED), tympanum diameter (TD), humerus length arm width (AW), forearm width (FW), forearm length (FAL), hand length (HAL), thigh length (THL), tibia length (TBL), tarsus length (TRL), and foot length (FL) (Table 1). The nomenclature and measurements follow Duellman (1970) except for the ESD, AW, FW, FAL, HAL and TRL which follow Carvalho-e-Silva, Silva & Carvalho-e-Silva (2009). Material examined in addition to the type series is listed in Appendix 1.

**Table 1:** Measurements (in millimeter) of the holotype and entire type series of *Phasmahyla lisbella* sp. nov. (the mean is followed by the standard deviation and ranges in parentheses).

Males										
*Phasmahyla lisbella* sp. nov. (*n* = 8)										
Measurements	ZUFMS-AMP 08803	ZUFMS-AMP 08804	ZUFMS-AMP 08806	MZUFV 18685	MZUFV 18686	ZUFMS-AMP 08808	ZUFMS-AMP 08809	ZUFMS-AMP 08811	Range	Mean ± SD
SVL	34.4	32.1	30.9	33.9	30.6	30.4	31.4	31.8	30.4–34.4	31.9 ± 1.5
HL	9.7	8.9	10.2	9.8	10.0	10.1	9.1	10.3	8.9–11.2	9.8 ± 0.5
HW	11.6	11.0	11.0	11.8	10.9	10.9	10.8	11.1	9.8–11.8	11.1 ± 0.4
IOD	5.1	4.1	4.7	4.6	4.5	5.2	4.1	4.5	4.1–5.2	4.6 ± 0.4
END	3.0	2.9	3.2	2.7	3.2	3.1	3.1	3.0	2.7–3.2	3.0 ± 0.2
ESD	4.5	3.9	4.3	4.7	4.7	4.9	4.4	4.5	3.9–4.9	4.5 ± 0.3
IND	2.1	1.8	2.2	2.4	2.2	2.0	1.7	2.2	1.7–2.4	2.1 ± 0.2
ED	4.3	4.6	4.6	4.1	3.8	4.4	3.3	3.9	3.3–4.6	4.1 ± 0.4
TD	1.6	1.9	1.7	1.6	1.6	1.8	1.6	1.6	1.6–1.9	1.7 ± 0.1
AW	1.6	1.6	1.0	1.5	1.4	1.2	1.1	1.6	1.0–1.6	1.4 ± 0.2
FW	3.0	2.5	2.1	2.4	2.3	2.1	2.4	2.2	2.1–3.0	2.4 ± 0.3
FAL	9.8	8.7	9.5	8.9	9.3	10.1	8.5	9.9	8.5–10.1	9.3 ± 0.6
HAL	7.5	8.7	7.0	6.9	7.4	7.5	6.9	7.7	6.9–8.7	7.4 ± 0.6
THL	15.9	14.8	16.1	15.5	15.2	15.2	15.0	16.9	14.8–16.9	15.6 ± 0.7
TL	16.8	15.1	16.1	15.8	15.5	16.6	14.9	16.8	14.9–16.8	15.9 ± 0.8
TRL	11.5	10.7	10.9	11.2	11.3	11.8	10.0	11.3	10.0–11.8	11.1 ± 0.6
FL	9.7	8.7	8.8	9.4	8.5	9.9	9.2	9.4	8.5–9.9	9.2 ± 0.5

**Note:**

Abbreviations are specified in “Materials and Methods.”

Twenty tadpoles from stage 26 to 42 (stages following Gosner, 1960) were used for the descriptions of the larval morphology. Tadpole measurements are as follows: total length (TL), body length (BL), ED, tail length (TAL), maximum tail height (MTH), tail muscle height (TMH), IND, IOD, tail muscle width (TMW), nostril-snout distance (NSD), oral disc width (ODW), END, body width (BW) and body height (BH). The nomenclature and measurements follow Altig & McDiarmid (1999), except for the ED, NSD, ODW, END, BW and BH which follow Randrianiaina et al. (2011).

### Molecular data

We sequenced fragments of 16S ribosomal RNA mitochondrial gene from two paratopotypes (ZUFMS-AMP 08808-09) of the new species. We extracted genomic DNA from muscle and/or liver samples using the phenol-chloroform protocol of Sambrook, Fritschi & Maniatis (1989). We used the 16Sa/16Sb primer pair of Palumbi et al. (1991), following PCR conditions described by Costa et al. (2016). PCR reactions consisted of 1 × buffer, dNTP at 0.2 mM, each primer at 0.2 μM, MgCl_2_ at 2 mM, 1 U Taq polymerase and 2 μl of template DNA, in a total reaction volume of 25 μl. We used the following PCR cycling program: 94 °C for 2 min, followed by 35 cycles of 94 °C for 30 s, 59 °C for 1 min, and 72 °C for 1 min, and concluding with a 5 min extension at 72 °C. We purified PCR products with Ethanol/Sodium Acetate and sequenced them on an ABI 3730XL DNA Analyzer (Applied Biosystems, Foster City, CA, USA). Resulting sequences were edited and aligned using Geneious 9.1.2 with the MUSCLE algorithm using default parameters (Edgar, 2004). The 16S mtDNA gene alignment present gaps, which were removed using GBLOCKS v.0.91b (Castresana, 2000; Talavera & Castresana, 2007), available online (http://molevol.cmima.csic.es/castresana/Gblocks_server.html). The final dataset, which was used for all analysis, has 331 base pairs (bp) for 16S.

For phylogenetic analyses, we based on 16S sequences from 21 specimens of *Phasmahyla* available in NCBI (*Phasmahyla cochranae*, *P. cruzi*, *P. exilis*, *P. guttata*, *P. jandaia* and *P. spectabilis*) available in GenBank (https://www.ncbi.nlm.nih.gov/genbank/) along with *Phyllomedusa camba*, *P. distincta*, *P. megacephala*, *P. rohdei* and *Boana alfaroi* as outgroups (Document S1). We also determined the model of nucleotide substitution for 16S with jModelTest (Darriba et al., 2012) using the Bayesian Information Criterion. The best-fit models was TrN+G. First, we performed a Bayesian phylogenetic analysis of 16S using BEAST v.1.8 (Drummond et al., 2012) for 20 million generations, sampling every 1,000 steps using a Yule Process tree prior. We checked for stationarity by visually inspecting trace plots and ensuring that all values for effective sample size were above 200 in Tracer v1.5 (Drummond & Rambaut, 2007). The first 10% of sampled genealogies were discarded as burn-in, and the maximum clade credibility tree with median node ages was calculated with TreeAnnotator v1.8 (Drummond et al., 2012). We also calculated sequence divergence (uncorrected *p*-distance) among species/individuals using MEGA v6.06 (Tamura et al., 2013). In order to explore the relationship among haplotypes, we estimated haplotype networks among species of *Phasmahyla* for the 16S mtDNA gene in POPART software (Leigh & Bryant, 2015) by using median-joining network method. We identified each species using different colors in the haplotype network.

### Nomenclatural acts

The electronic version of this article in Portable Document Format (PDF) will represent a published work according to the International Commission on Zoological Nomenclature (ICZN), and hence the new names contained in the electronic version are effectively published under that Code from the electronic edition alone. This published work and the nomenclatural acts it contains have been registered in ZooBank, the online registration system for the ICZN. The ZooBank Life Science Identifiers (LSIDs) can be resolved and the associated information viewed through any standard web browser by appending the LSID to the prefix http://zoobank.org/. The LSID for this publication is: urn:lsid:zoobank.org:pub:DAD55134-8943-4189-AF4A-7004FA5FCD51. The online version of this work is archived and available from the following digital repositories: PeerJ, PubMed Central, and CLOCKSS.

## Results

**Figure 1:**
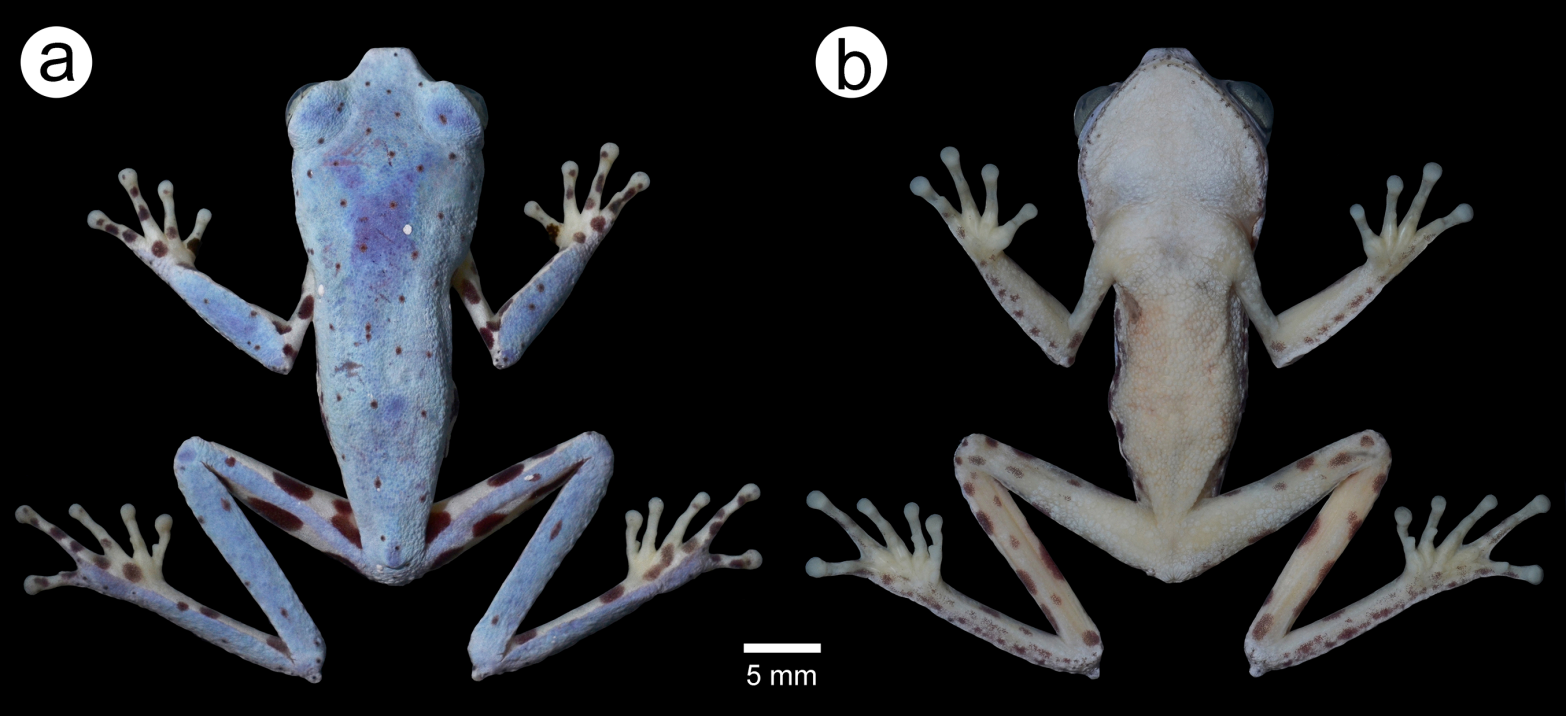
Holotype of *Phasmahyla lisbella* (ZUFMS-AMP 8803). (A) Dorsal view of the body; (B) ventral view of the body. Image credit/source: Francisco Severo Neto.

**Figure 2:**
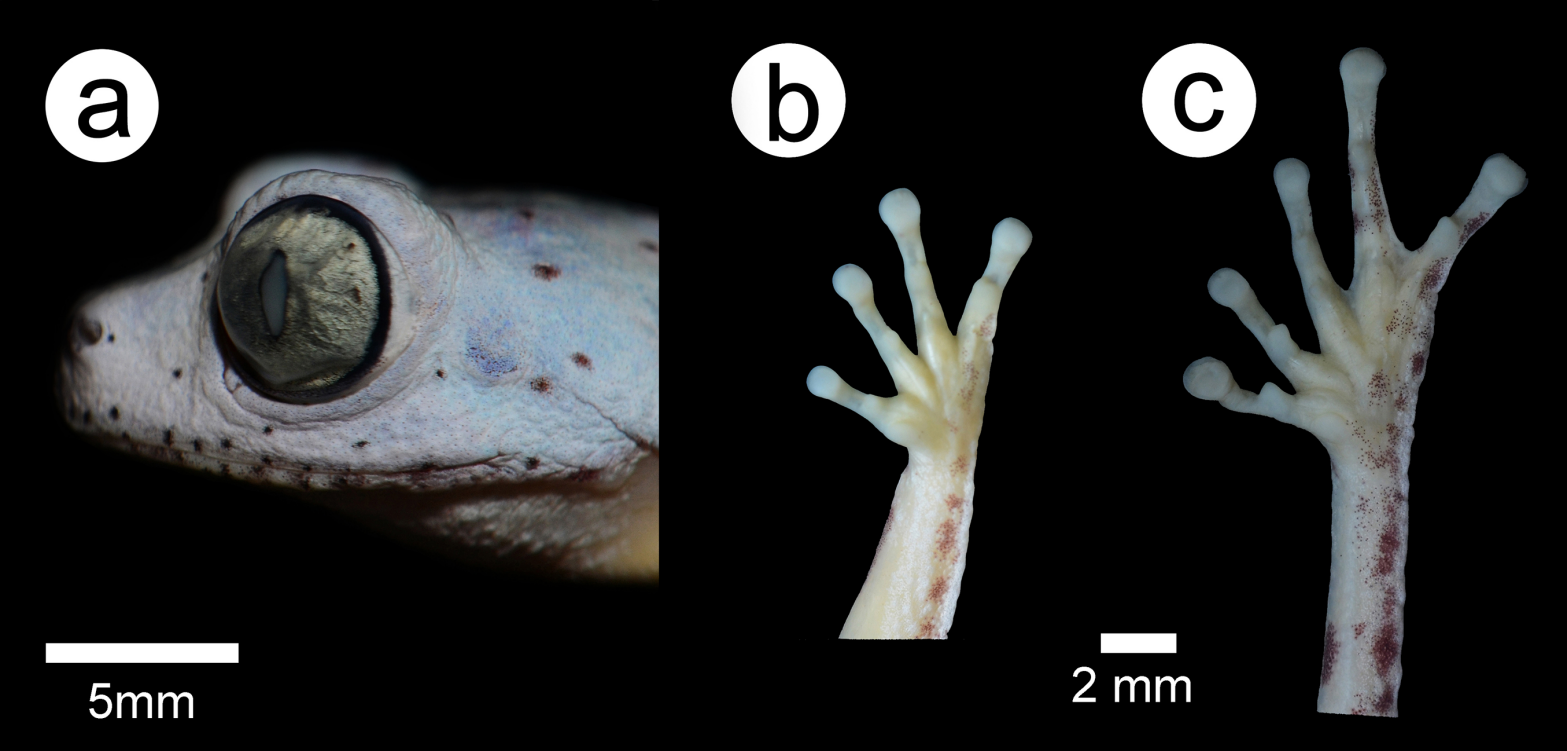
Holotype of *Phasmahyla lisbella* (ZUFMS-AMP 8803). (A) Head lateral views; (B) ventral view of right hand; and (C) ventral view of right foot. Scale bars = 5 mm. Image credit/source: Francisco Severo Neto.

**Figure 3:**
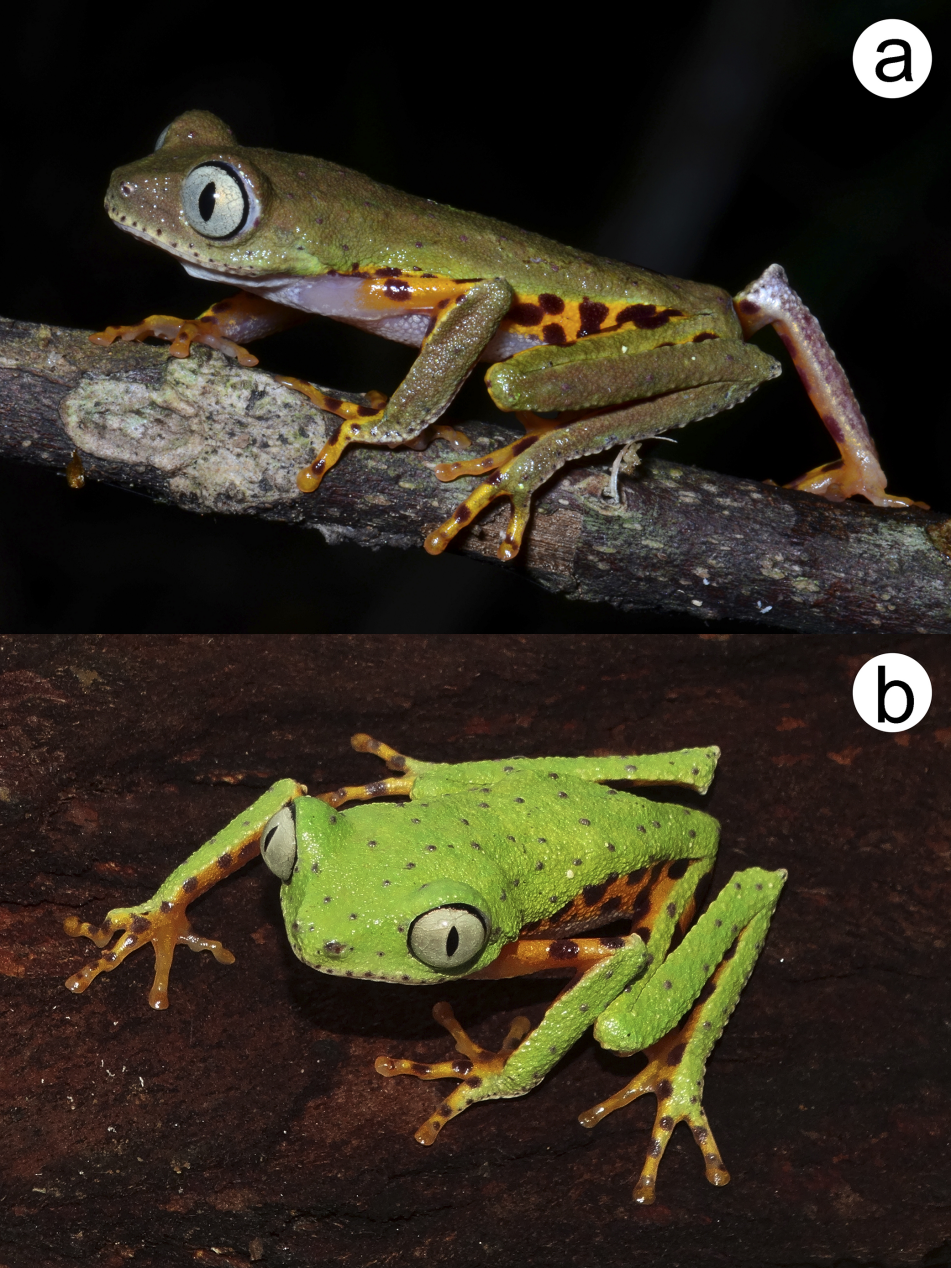
*Phasmahyla lisbella* sp. nov. in life from the type locality (ZUFMS-AMP 8803). (A) Nocturnal and (B) diurnal coloration. Image credit/source: (A) D.J. Santana and (B) by H. Folly.

*Phasmahyla lisbella* sp. nov. (Figs. 1–3; Table 1).

ZooBank LSID: urn:lsid:zoobank.org:act:48E90F44-394E-4C53-BEDA-0230585524A8.

### Holotype

ZUFMS-AMP 08803, adult male, from the Fazenda Ventania (21°20′7.62″S, 42°12′15.40″W, 536 m above sea level), located in the Área de Proteção Ambiental Ventania, Miracema municipality, Rio de Janeiro state, Brazil, collected by D. J. Santana, H. Folly and L.C.L. Rocha on 24 October 2017.

### Paratopotypes

Four adult males (ZUFMS-AMP 8804-06; MZUFV 18685) collected along with the holotype by the same collectors; four adult male (ZUFMS-AMP 8808-09, 8811; MZUFV 18686) from the type locality, collected by L.C.L. da Rocha on 14 September 2017.

### Diagnosis

The new species, *P. lisbella*, is characterized by: (1) grainy dorsal skin; (2) calcar well developed and broad at the base; (3) presence of rounded purple patches in hidden areas of the arm, forearm, thigh, tibia, tarsus, and toes; (4) inner parts of legs and flanks orange colored, with numerous round violet blotches in life; (5) reduced laterodorsal glands; (6) *Canthus rostralis* slightly distinct; (7) eyes large, and the palpebral membranes translucent over their entire area; (8) forearms slender in males; (9) tarsus large, outer margin smooth or slightly crenulated; (10) fingers medium sized; (11) advertisement call composed by one pulsed note with only one pulse; (12) tadpoles with oral disc large and wide, with a deep recess in the dorsal margin and a less sharp recess in the ventral margin; (13) tadpoles with tooth row formula 0/2(1).

### Comparisons with other species

*Phasmahyla lisbella* sp. nov. differs from *P. cochranae* by *C. rostralis* slightly distinct (well marked, view from above very concave in *P. cochranae*) and calcar developed (poorly developed in *P. cochranae*). The tadpoles of *P. lisbella* sp. nov. differ from those of *P. cochranae* because they do not have row of teeth in the anterior part (row A1 with teeth in *P. cochranae*: Bokermann, 1966).

The new species differs from *P. exilis* by the ornamentation with purple drops on flanks and concealed surfaces of forearms, thighs, and digits (reduced and only present on flanks and concealed surfaces of thighs in *P. exilis*), concealed surface of limbs ornamented with numerous small rounded purple spots (absence of ornamentation in the concealed surfaces of limbs in *P. exilis*), and the body more robust than *P. exilis*. The tadpoles of *P. lisbella* sp. nov. differ from those of *P. exilis* because they do not have row of teeth in the anterior part (row A1 with teeth in *P. exilis*: Cruz, 1980).

*Phasmahyla lisbella* differs from *P. jandaia* by having calcar developed (weakly developed in *P. jandaia*). The tadpoles of *P. lisbella* sp. nov. differ from those of *P. jandaia* because they do not have row of teeth in the anterior part (row A1 with teeth in *P. jandaia*: Bokermann & Sazima, 1978).

The new species differs from *P. spectabilis* by having a calcar well developed (a little developed in *P. spectabilis*), by its advertisement call (1 pulse in *P. lisbella* sp. nov., 2–4 pulses in *P. spectabilis*), and by concealed surface of limbs ornamented with numerous small rounded purple spots (moderate ornamentation with purple drops on flanks and concealed surfaces of forearms, thighs and digits). The tadpoles of *P. lisbella* sp. nov. differ from those of *P. spectabilis* because they do not have row of teeth in the anterior part (row A1 with teeth in *P. spectabilis*: Cruz, Feio & Nascimento, 2008).

*Phasmahyla lisbella* differs from *P. timbo* by the presence of rounded purple patches in hidden areas of the arm, forearm, thigh, tibia, tarsus, and toes, which are missing or very faint in *P. timbo*; by its reduced laterodorsal glands, which are well developed in *P. timbo* and by advertisement call (1 pulse in *P. lisbella* sp. nov., 2–4 pulses in *P. timbo*). The tadpoles of *P. lisbella* sp. nov. differ from those of *P. timbo* because they do not have row of teeth in the anterior part (row A1 with teeth in *P. timbo*: Cruz, Napoli & Fonseca, 2008).

The new species is distinguished from *P. guttata* by the forearms slender in males (robust in *P. guttata*), by fingers medium sized (large in *P. guttata*). The tadpoles of *P. lisbella* sp. nov. differ from those of *P. guttata* because they do not have row of teeth in the anterior part (row A1 with teeth in *P. guttata*: Lutz, 1924).

The new species differs from *P. cruzi* by the grainy dorsal skin (smooth in *P. cruzi*); by palpebral membrane translucent (reticulated in *P. cruzi*) and by the length of tarsus and foot/SVL (63% and 68.4% respectively). In tadpoles, differs from *P. cruzi* by the shape of the anterior end of the oral disc (slightly wrapped in *P. cruzi*: Carvalho-e-Silva, Silva & Carvalho-e-Silva, 2009; strongly wrapped in *P. lisbella*).

Additionally, *P. lisbella* also differs from most of its congeners by differences in tadpole external morphology and morphometry (see below).

### Description of holotype

A medium size specimen (SVL = 34.4 mm, *n* = 8); skin grainy; slender body and limbs; HW about 34% of SVL; head wider than longer (HL about 83% of its width; snout short, truncated in dorsal view, vertical and slightly rounded in profile, ESD equal to 21% of HL and 5% of SVL; *C. rostralis* slightly distinct; loreal region plane; nostrils located at end of snout and directed towards sides; IND equal to 18% of HW; eyes large, protuberant, directed anterolaterally, their diameter equal to 37% of HW; vertical pupil; palpebral membrane translucent; tympanum small, hidden above by the supratympanic fold, only the lower part visible, its diameter corresponding to 17% of HL; vomerine teeth and vocal slits absent, tongue rounded and not notched in posterior area.

Arms thin, equal in width to 4% of SVL; FW equal to 8% of SVL, internal margins of forearm slightly crenulated; dermal ridges at elbows; fingers thin, free, with no web, and with small discs on the tips, finger length order I<II<IV<III, nuptial pads formed by the union of very small brown horny asperities on the base of first finger; subarticular tubercles round and prominent, carpal tubercle ovoid. Hind limbs slender, with a distinct calcar; the sum of the thigh and tibia lengths equal to 94% of SVL, tibia borders crenulated forming a fringe; tarsus large, with crenulated external borders; the sum of the tarsus and FLs equal to 61% of the SVL; toes long and slender with small discs on their tips, length order: I<II<III<V<IV, subarticular tubercle rounded and prominent, inner metatarsal tubercle ovoid and moderate; outer metatarsal tubercle absent.

### Coloration of holotype in preservative

Dorsal surfaces of body, forearms, legs, loreal, and tympanic regions purple, with rounded spot dark brown scattered; upper arms, fingers, and toes cream with small purple drops on all fingers, toes II, IV and V; iris gray; flanks, inguinal region, internal and external sides of tibia, and internal side of tarsus cream with sparse purple drops; ventral surfaces cream; nuptial asperities cream.

### Measurements of holotype

SVL = 34.37; HL = 9.67; HW = 11.61; IOD= 5.09; END = 3; ESD= 4.5; IND = 2.05; ED = 4.29; TD = 1.61; AW = 1.64; FW = 2.96; FAL = 9.82; HAL = 7.51; THL = 15.90; TL= 16.8; TRL= 11.45; FL = 21.21.

### Color of holotype in life

Dorsal surfaces of body, forearms, legs, loreal, and tympanic regions moss green with small purple drops and slightly grainy light brown skin; upper arms, fingers, and toes orange with small purple drops on fingers IV, toes IV and V; iris silver-gray; palpebral membrane transparent; flanks, inguinal region, internal and external sides of tibia, and internal side of tarsus orange with numerous small rounded purple spots; ventral surfaces whitish; nuptial asperities dark brown (Figs. 3A and 3B).

### Variation

Examined specimens are congruent with respect to the morphological characters and color. The sum of thigh and TL varies from 90% to 110% of SVL. Variations in measurements are presented in Table 1.

### Advertisement call

**Figure 4:**
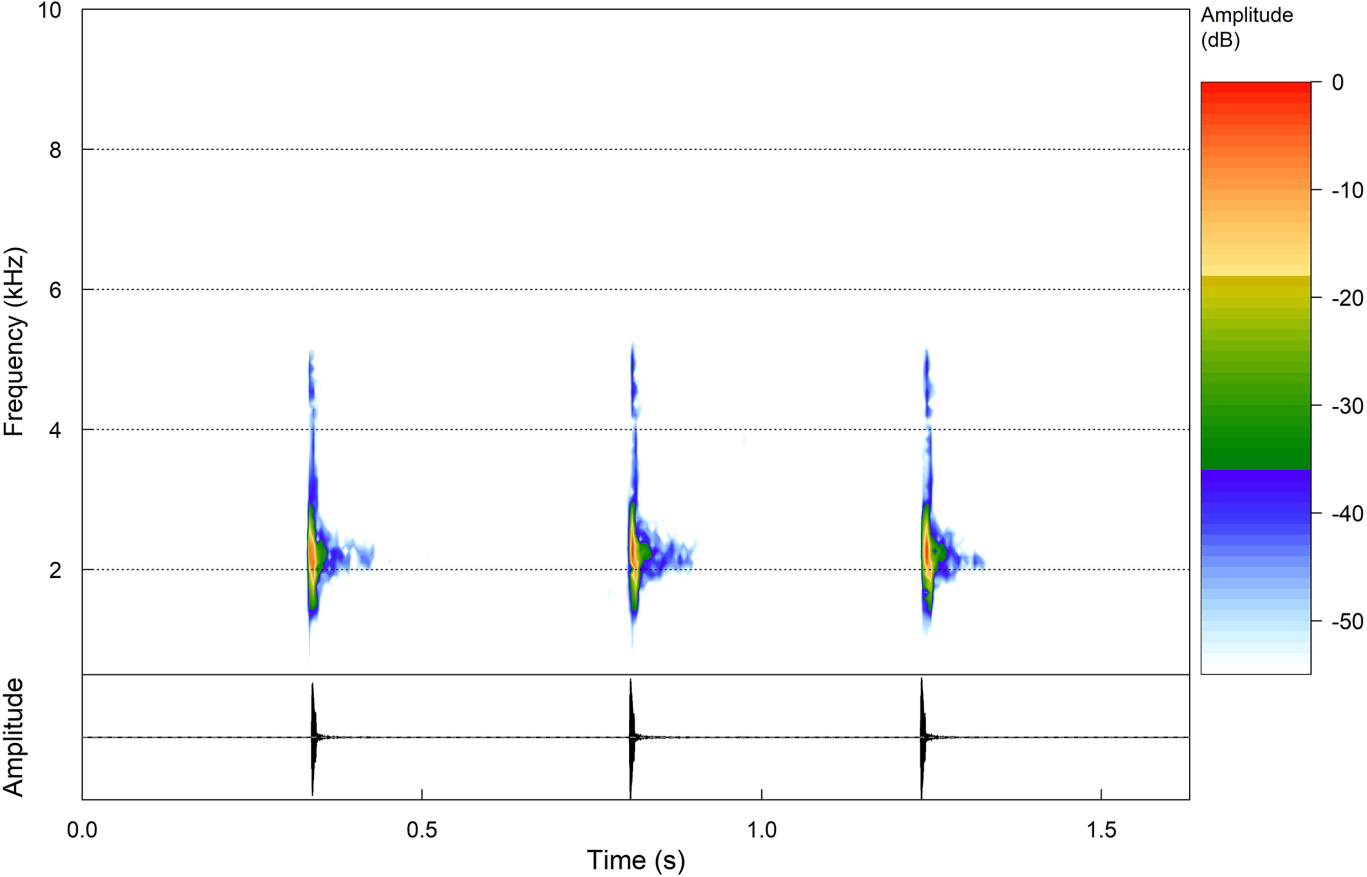
Advertisement calls. Oscilogram and Spectogram of a sequence of three advertisement calls of the holotype of *Phasmahyla lisbella* sp. nov. (ZUFMS-AMP08803) from the type locality (water temperature 21.3 °C).

Three males were recorded (ZUFMS-AMP08803, *n* = 9), ZUFMS-AMP08804, *n* = 21) and (ZUFMS-AMP08806, *n* = 6). These calls were recorded in situ with an Tascam DR-40. Digital recordings were sampled at 96 kHz and 24 bit resolution and saved in uncompressed WAV format. Calls emitted sporadically and with one note, comprised by one pulse (Fig. 4). Call (= note) duration ranged from 0.007 to 0.087 s (0.039 ± 0.021; *n* = 36), and the call dominant frequencies ranged from 1894.9 to 2239.5 Hz (2100.7 ± 107.6; *n* = 36). Calls without frequency modulation (Table 2).

**Table 2:** Advertisement calls described for the genus *Phasmahyla*.

Call Parameter	*P. spectabilis* (Dias et al., 2011)	*P. timbo* (Cruz, Napoli & Fonseca, 2008)	*P. lisbella*	*P. lisbella*	*P. lisbella*
			ZUFMS-AMP08803 (Holótipo) (*n* = 9)	ZUFMS-AMP08804 (Parátipo) (*n* = 21)	ZUFMS-AMP08806 (Parátipo) (*n* = 6)
Dominant Frequency (Hz)	1,687–1,907	1,560–1,940	1,894.9–2,239.5	1,894.9–2,239.5	2,067.2
	(1,849 ± 79.05)	(1,750 ± 100)	(2,143.8 ± 125.2)	(2191.9 ± 110.1)	
Call Duration (s)	0.021–0.057	0.08–0.09	0.007–0.028	0.040–0.087	0.011–0.013
	(0.039 ± 0.009)	(0.027 ± 0.004)	(0.019 ± 0.006)	(0.055 ± 0.009)	(0.012 ± 0.001)
Pulse Number	2–4	2–4	1	1	1
Pulse Duration (s)	0.002–0.014	0.0037–0.0063	–	–	–
	(0.0063 ± 0.0026)	(0.0037 ± 0.0006)			
Pulse Intervall (s)	0.002–0.013	0.005–0.011	–	–	–
	(0.092 ± 0.0019)	(0.0089 ± 0.0012)			

**Notes:**

Values are presented as range (mean ± SD).

SD, standard deviation.

### Tadpole description (stage 37)

**Figure 5:**
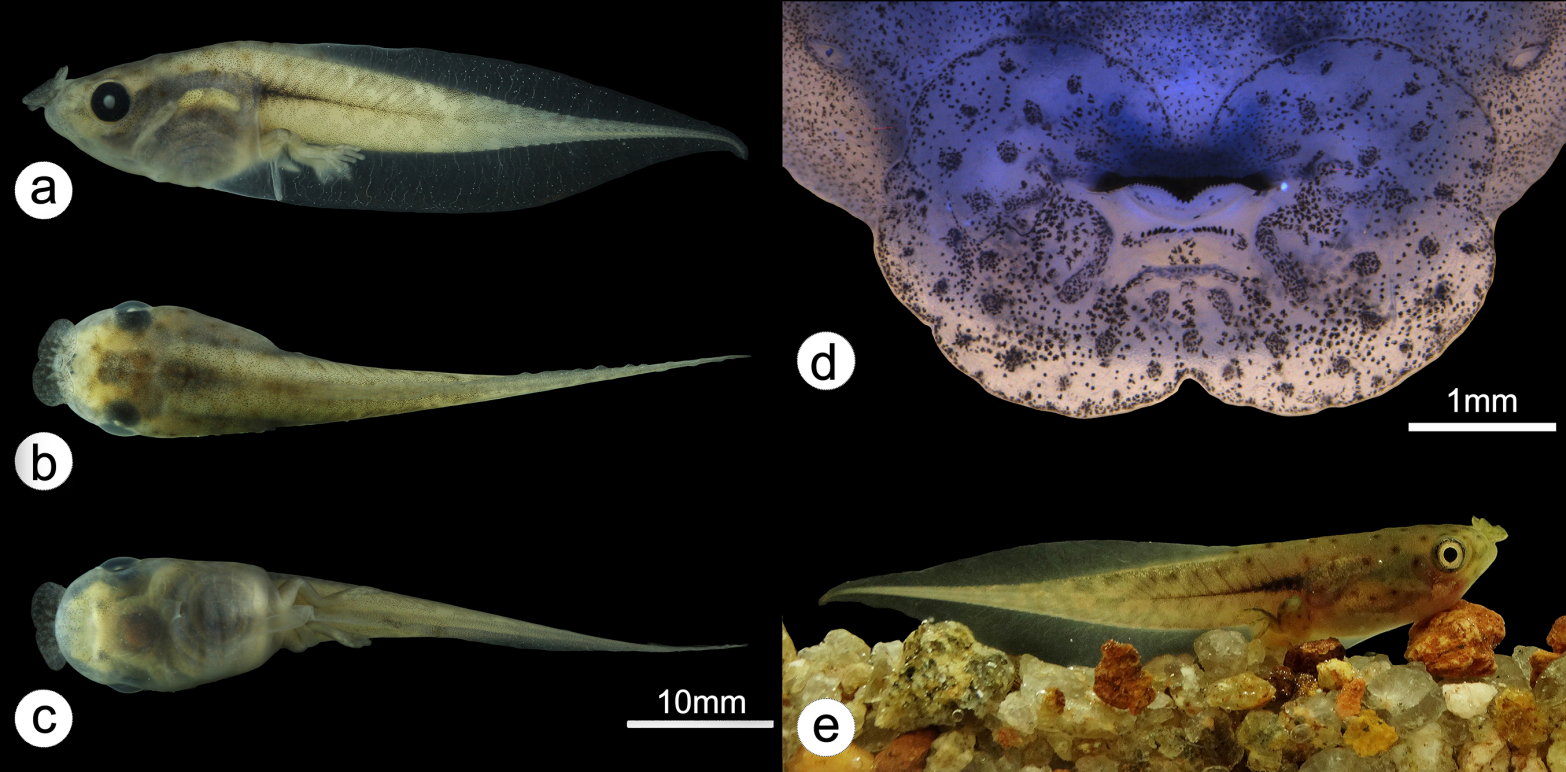
Tadpole. (A) Tadpole (stage 37) in lateral view; (B) in dorsal view; (C) in ventral view; (D) oral disc and (E) tadpole (stage 39) in life (ZUFMS-AMP08879). Image credit/source: (A, B, C and E) H. Folly and (D) D.J. Santana.

(ZUFMS-AMP08879) SVL = 41.3–44 mm, body ovoid in dorsal view, broad, anterodorsal mouth with funnel-shaped dermal fold; eyes directed laterally, well developed, separated from each other by 63% of BW, their diameters equal to 35% of BW; nostrils weakly distinct, ovoid, longer than wide, placed laterally on body, separated from each other by 69% of BW; IND slightly larger than interocular distance; spiracle in the middle of body, ventral, slightly sinistral, not protruding, with widely visible opening, appearing to be dug into the body wall; anal tube long, free, right of ventral fin; TAL corresponding to 67% of total length and its height equal to 36% of its length; tail narrow, with well-developed musculature, ending in flagelliform tip, generally curved toward the bottom; dorsal fin low, 26% of tail height; dorsal fin originating slightly end of body and remaining linear until mid-tail area, gradually diminishing in height towards tip of tail; ventral fin higher than dorsal fin, 39% of tail height (Figs. 5A–5C).

Oral disc modified as large funnel-shaped anterodorsal structure 4.6 mm wide, with a deep recess in the dorsal margin and a less sharp recess in the ventral margin and the lateral margins may be slightly emarginated or not emarginated (Fig. 5D); one series of small papillae on internal face of oral disc, and its border surrounded by many smaller papillae, with two larger and elongated papillae on each side of horny beak; many medium-sized ovoid papillae spread over entire surface; upper and lower jaw sheaths with finely serrate margins, upper jaw with conical projection, lower jaw V-shaped; tooth row formula 0/2(1) and rarely 0/2; generally no horny denticle above horny beak and with two rows below, first (P1) with medial gap and second (P2) continuous and rarely first (P1) continuous too, narrower than P1 (Fig. 5D).

### Tadpole coloration in life (stage 39)

The tadpole in life has a beige-green dorsal with sparse rounded spots. It has belly and extremity of the tail muscles lighter than the pigmentation of the body. The caudal musculature is light brown pigmented with undefined spots of beige color and has a dark median longitudinal band completely reaching the first third of the tail muscles. The pupil of the eye is black and the iris is creme (Fig. 5E).

### Preserved tadpole coloration

The preserved tadpole is grayish-beige, with dark brown spots concentrated in the cephalic and lateral region of the head. The caudal musculature is light brown pigmented with undefined spots of beige color and has a dark median longitudinal band completely reaching the first third of the tail muscles. The belly is translucent, with light brown pigmentation and the fin is transparent (Figs. 5A–5C).

### Measurements of tadpoles in stage 37 (*n* = 4, in mm)

Mean ± standard deviation and range.

TL: 42.5 ± 1.3 (41.28–43.97); BL: 13.4 ± 1 (12.1–14.56); ED: 2.5 ± 0.1 (2.39–2.63); TAL: 28.8 ± 1.2 (27.11–29.87); MTH: 10.4 ± 1.1 (9.26–11.44); TMH: 3.4 ± 0.3 (3.15–3.69); IND: 4.9 ± 0.2 (4.63–5.08); IOD: 4.5 ± 0.3 (4.08–4.71); TMW: 4.1 ± 0.3 (3.67–4.31); NSD: 3.3 ± 0.2 (3.18–3.47); ODW: 4.6 ± 0.4 (3.98–4.86); END: 1.3 ± 0.2 (1.09–1.46); BW 7 ± 0.6 (6.23–7.6); BH 8.3 ± 0.8 (7.2–9.21). Measurements of tadpoles for other stages are summarized in Table 3.

**Table 3:** Tadpoles mean measurements (mm) of *Phasmahyla lisbella* sp. nov.*, P. cruzi, P. guttata, P. cochranae, P. exilis, P. jandaia, P. spectabilis,* and *P. timbo* at stages 36 or 37 (Gosner, 1960).

Species/measurements	TL	BL	ED	TAL	MTH	TMH	IND	IOD	TMW	NSD	ODW	END	BW	BH
*Phasmahyla lisbella* sp. nov. (stage 37) (*n* = 4)	42.5	13.4	2.5	28.8	10.4	3.4	4.9	4.5	4.1	3.3	4.6	1.3	7	8.3
*Phasmahyla cruzi*^1^ (stage 37) (*n* = 10)	42.80	14.20	2.10	28.70	7.70	–	6.40	6.70	–	–	7.00	1.20	7.10	6.80
*Phasmahyla guttata* (stage 37) (*n* = 5)	49.40	15.80	1.80	33.60	8.50	–	6.60	8.00	–	–	6.10	2.20	8.70	7.90
*Phasmahyla cochranae*^2^ (stage 36) (*n* = 1)	43.00	16.00	2.50	27.00	–	–	6.00	7.00	–	–	–	2.00	9.50	10.00
*Phasmahyla exilis*^2^ (stage 37) (*n* = 1)	39.50	13.00	2.50	26.50	–	–	5.50	6.50	–	–	–	1.00	8.00	7.50
*Phasmahyla jandaia*^2^ (stage 37) (*n* = 1)	42.50	14.00	2.50	28.50	–	–	6.00	6.50	–	–	–	1.50	8.00	7.50
*Phasmahyla spectabilis*^3^ (stage 36) (*n* = 1)	45.00	14.70	2.70	30.30	6.90	–	5.60	5.30	–	–	6.90	2.00	6.90	7.90
*Phasmahyla timbo*^4^ (stage 37) (*n* = 1)	39.00	14.00	2.40	25.00	10.40	–	5.60	5.00	–	–	6.20	1.40	8.00	8.70

**Notes:**

Abbreviations are specified in “Materials and Methods.”

1 Carvalho-e-Silva, Silva & Carvalho-e-Silva (2009).

2 Cruz (1982).

3 Cruz, Feio & Nascimento (2008).

4 Cruz, Napoli & Fonseca (2008).

### Phylogenetic analysis

**Figure 6:**
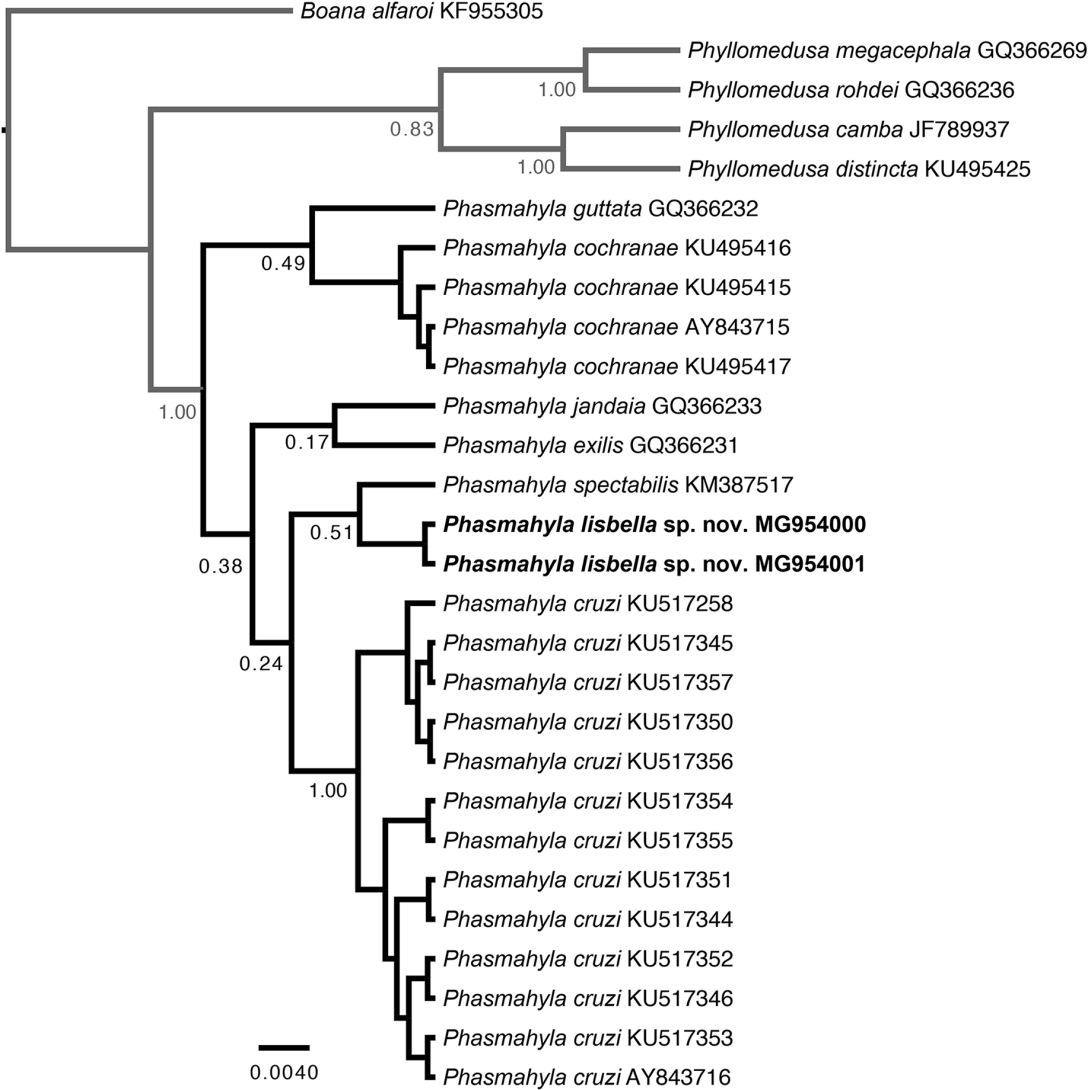
Gene tree. Tree topology based only on the 16S mDNA for six species of the genus *Phasmahyla*.

**Figure 7:**
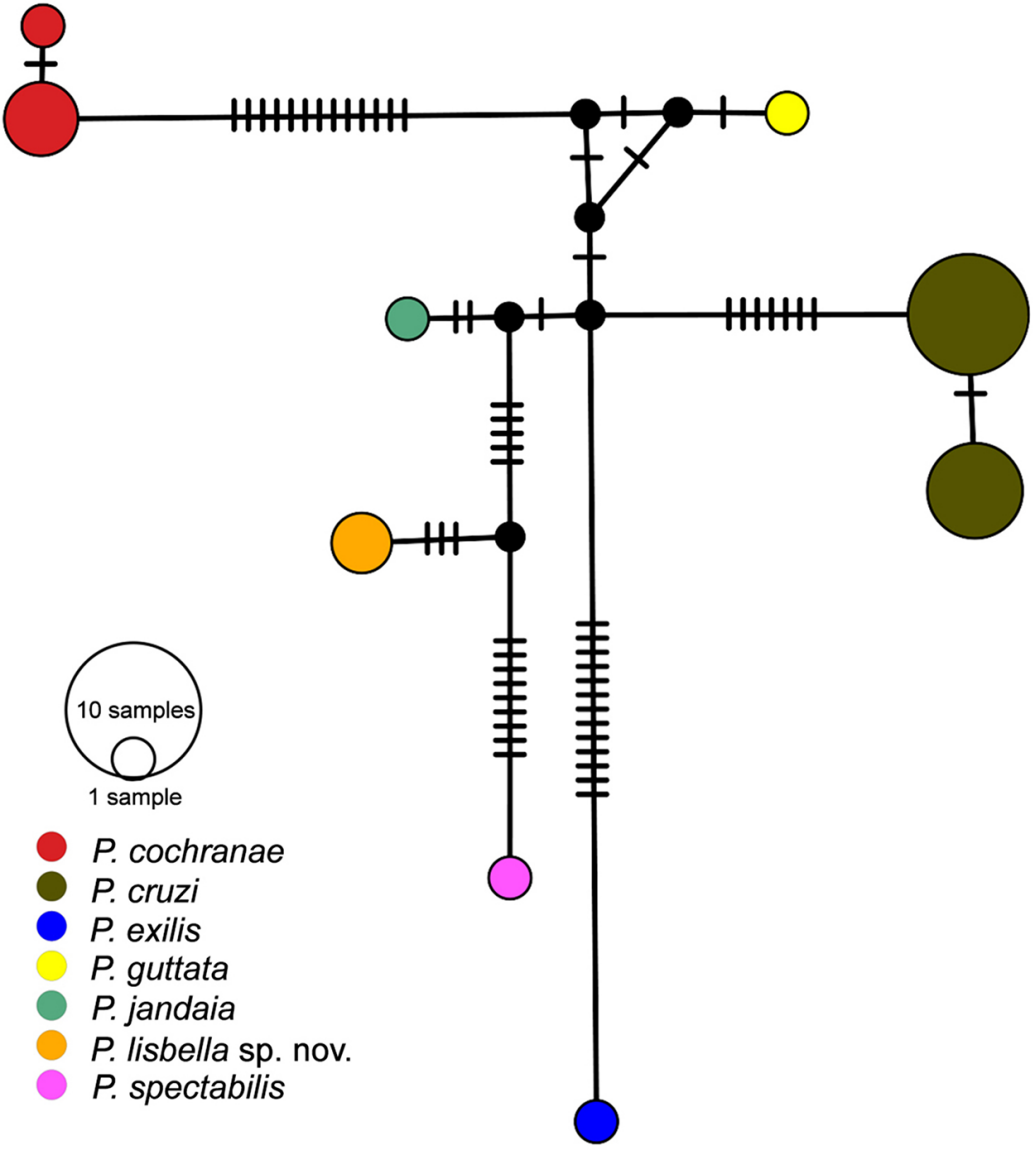
Median-Joining haplotype networks with base on the attribution of specimens on the basis of their mtDNA (16S) haplotype. Each haplotype is represented by a circle whose area is proportional to its frequency. Traits indicate additional mutational steps for branches with more than one mutation. The colors indicate the species-level units. The black dots are median vectors (hypothesized sequences).

Sequences of the mitochondrial 16S ribosomal RNA gene of two paratypes (ZUFMS-AMP 08808, ZUFMS-AMP 08809) of *P. lisbella* sp. nov. have been deposited at GenBank (MG954000, MG954001). Our tree topology, based only on the 16S mDNA recovered weakly posterior probabilities support values among lineages (Fig. 6). However, placed the new species as sister taxa of *P. spectabilis.* The mitochondrial haplotype network (Fig. 7) based on a fragment of the 16S rRNA gene (331 bp) shows nine distinct mitochondrial lineages and no haplotype sharing between any *Phasmahyla* species, neither between *P. lisbella* and *P. spectabilis* with 12 mutational steps between them (Fig. 7). Average sequence divergences between the new species and congeners range from 3.0% (*P. jandaia*) to 6.0% (*P. cochranae* and *P. exilis*) (Document S2).

### Habitat and natural history

We found individuals of *P. lisbella* sp. nov. in a fragment of the semideciduous seasonal forest of the Atlantic Forest domain, Miracema municipality, Rio de Janeiro state, Brazil. The new species is apparently common in the area and is easily found on the bushes inside the forest. The males were calling from 19.00 h to 22.00 h perched on herbaceous and shrubby vegetation between ∼20 and 1.75 cm above the ground of a stream in the interior of the forest. We also found tadpoles and a spawn along the same stream. *Ololygon flavoguttata* (Lutz & Lutz, 1939), *Phitecopus rohdei* (Mertens, 1926)*, Rhinella ornata* (Spix, 1824) were found sympatrically with *P. lisbella* sp. nov.

**Figure 8:**
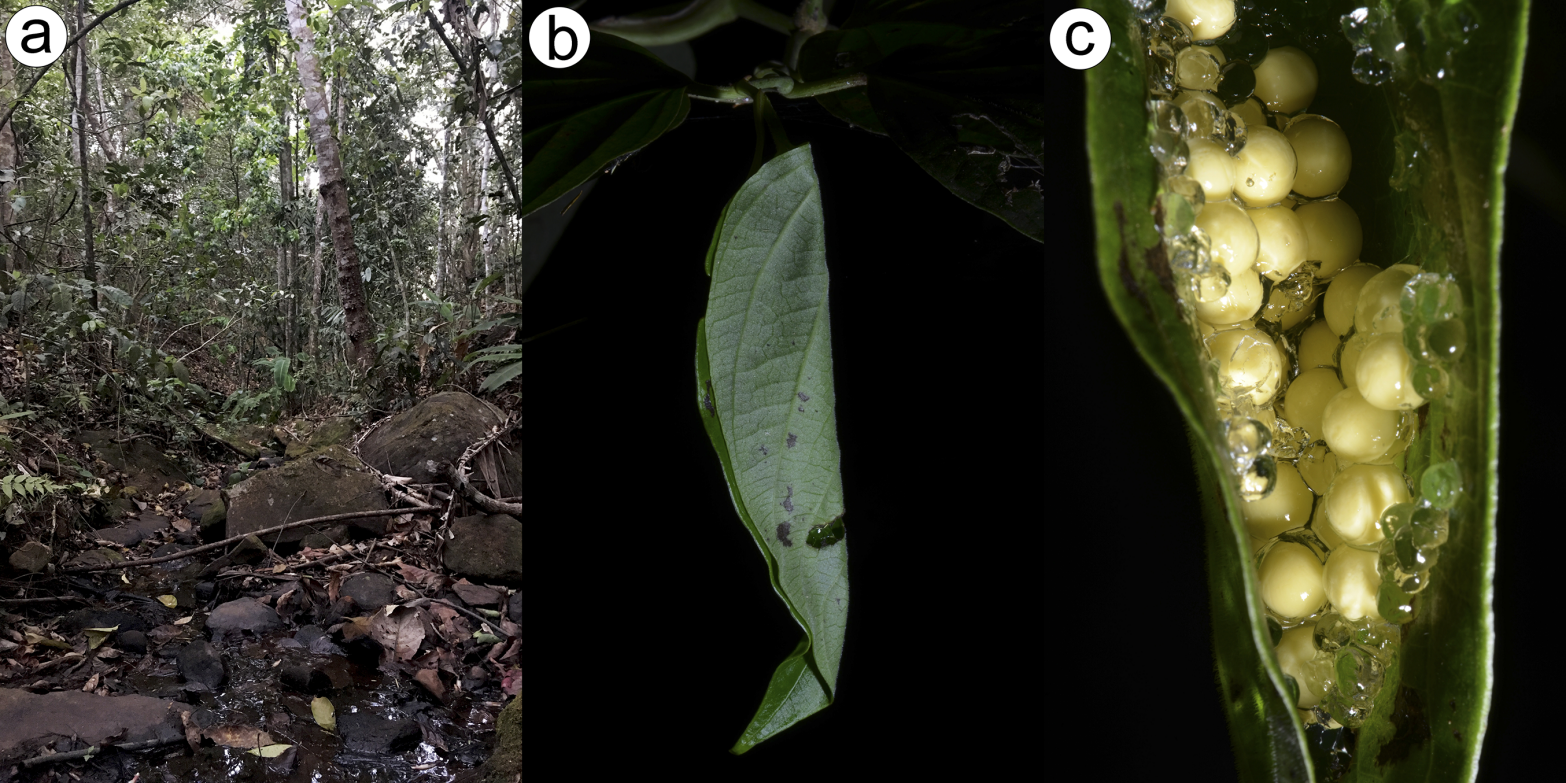
Habitat. (A) Habitat where the specimens was found; (B) Melastomataceae leaf; (C) Egg clutch with transparent jelly found on the leaf of the family plant Melastomataceae. Image credit/source: D.J. Santana.

We found 43 egg in the egg clutch observed on October 2017 at Fazenda Ventania, Área de Proteção Ambiental Ventania, Miracema municipality, Brazil. Each egg is cream colored, and the spawn was found in the adaxial parts of the leaf, approximately 1 m above water. The egg clutch was deposited in a laminar array with transparent jelly (Figs. 8A–8C).

### Distribution

**Figure 9:**
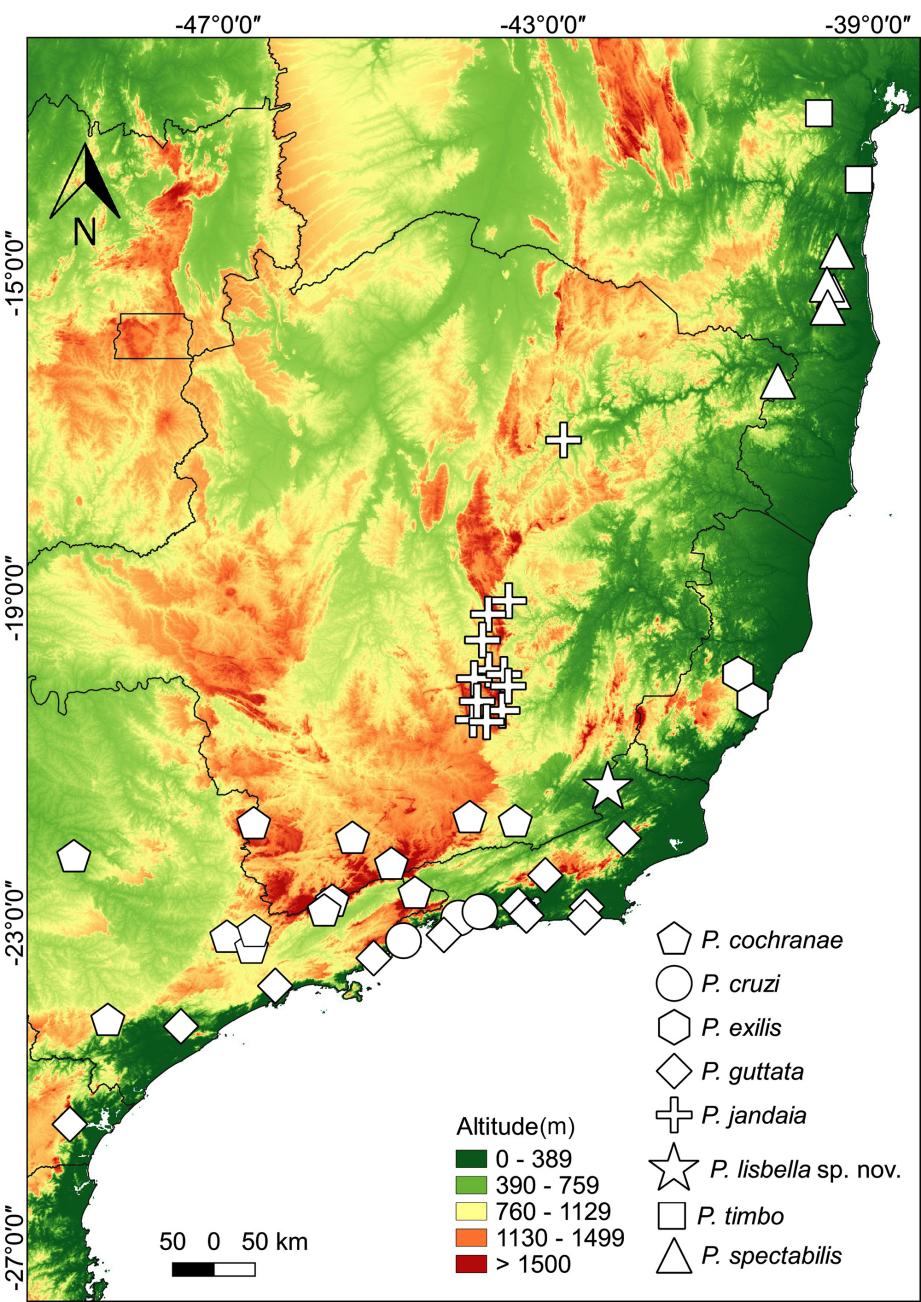
Distribution map of Phasmahyla species in Atlantic Forest. Geographic distribution of Phasmahyla in the Atlantic Forest, including those of the new species. Gray area represents the limits of the Atlantic Forest.

The new species is known only from its type locality (Fazenda Ventania, Área de Proteção Ambiental Ventania, Miracema municipality, Rio de Janeiro state, Brazil) (Fig. 9).

### Etymology

The specific name is a noun, honoring Lis Alves Pereira de Oliveira da Rocha and Bella Alves Pereira Custódio da Rocha, nieces of L.C.L. Rocha. Citizens of Miracema, and future representatives for nature conservancy in the region.

## Discussion

The use of colors as a defensive strategy by anurans may be widespread, and its protective effect may vary depending of the species (Toledo & Haddad, 2009). These colors could be described as camouflage, where an animal blends with part of its environment, such as branches and leaves. Although this change in color pattern is known for phyllomedusines as polyphenism (*sensu* Hanlon, Forsythe & Joneschild, 1999). It was described as change from purplish during night activity to greenish during daytime resting (Toledo & Haddad, 2009) for *P. cochranae* (Bokermann, 1966), *P. guttata* (Machado et al., 2015), *P. jandaia* (Bokermann & Sazima, 1978) displaying a change of color from brown to green. We observed the same color changing for this new species (Fig. 3).

It is reported that *Phasmahyla guttata* tadpoles undergo ontogenetic variations in development (Costa & Carvalho-e-Silva, 2008). We verify such variation in *P. lisbella* sp. nov. Among the changes, the buccal funnel grew until stage 41, and from there began to be absorbed. The tail grew until stage 41, and in the 42 began to regress. IOD increased from stage 26 to stage 39 and after this stage began to decrease. The IND increased until stage 37 and from there began to decrease. The distance between the eye and the nostril grew to stage 37 and remained almost constant after this stage. In order to compare species, we also recommend the use of tadpoles that are in the same stage of development, usually stage 37.

Unfortunately, most of the natural habitats of the Área de Proteção Ambiental Ventania are severely fragment and degraded by unsustainable timber extraction, uncontrolled expansion of the agriculture, and replacement by non-native plantations. Conservation of the biodiversity and natural resources of the APA Ventania are mostly dependent on urgent conservation actions to preserve the last remnants of these diverse and unique forests. We highlight the urgent need of development for research and conservation actions in the near future, in order to improve knowledge and promote conservation of *P. lisbella* sp. nov. and consequently, the biodiversity of the region. Surveying other areas to determine the extent of occurrence of *P. lisbella* sp. nov. and other range-restricted species is imperative to proper understanding how threatened the new species might be.

## Appendix 1

Material examined in addition to the new species.

*Phasmahyla cochranae*: Lambari-MG (MZUFV 8399-00), Parque Estadual do Ibitipoca-MG (MZUFV 2304), Poços de Caldas-MG (MZUFV 8173), Serra Negra-MG (MZUFV 15044), Bertioga-SP (UFMG-AMP 1564).

*Phasmahyla cruzi*: Ubatuba-SP (CFBH 39151), Itaguaí-RJ (RU-GIR 70).

*Phasmahyla exilis*: Cariacica-ES (CFBH 4022), Reserva Biológica de Duas Bocas-ES (MBML 4859).

*Phasmahyla guttata*: Rio de Janeiro-RJ (ZUEC-AMP 3378-79, RU-GIR 69), Ubatuba-SP (CFBH 05704).

*Phasmahyla jandaia*: Congonhas-MG (MZUFV 14550), Ouro Branco-MG (MZUFV 7561, 8481), Ouro Preto-MG (MZUFV 6031, 6033, 10347), Jaboticatubas-MG (ZUEC-AMP 3357), Serra do Gandarela–Fazenda Serra do Maquiné, Caeté-MG (UFMG-AMP 19641).

*Phasmahyla timbo*: Serra do Timbó-BA (MZUESC 16609), Igrapiúna-BA (MZUESC 16631, 16626, RU-GIR 71).

*Phasmahyla spectabilis*: Salto da Divisa-MG (MZUFV 4160), Parque Nacional do Alto do Cariri (Guaratinga)-BA (MZUESC 16602, 16616, 16622).

## Supplemental Information

10.7717/peerj.4900/supp-1 Supplemental Information 1 Document S1. List of sequences from GenBank used in this study (16S rRNA).

10.7717/peerj.4900/supp-2 Supplemental Information 2 Document S2. Uncorrected p-distances for a 331-bp aligned sequence of the 16S rRNA gene of the new species, 21 samples of other Phasmahyla species, one sample of Boana species and four samples of Phyllomedusa species taken from GenBank (see Document S1).

10.7717/peerj.4900/supp-3 Supplemental Information 3 Document S3. Sequences of the two paratypes of *Phasmahyla lisbella* sp. nov.

10.7717/peerj.4900/supp-4 Supplemental Information 4 Figure S1. Calcar appendages from all eight species of *Phasmahyla*. a) *P. cochranae* (UFMG-AMP 1564); b) *P. cruzi* (CFBH 39151); c) *P. exilis* (CFBH 4022); d) *P. guttata* (CFBH 05704); e) *P. jandaia* (UFMG-AMP 19641); f) *P. lisbella* sp. nov. (ZUFMS-AMP 08803, holotype); g) *P. spectabilis* (MZUESC 16616); h) *P. timbo* (MZUESC 16609, topotype). Image credit/source: (a and e) Sofia Velasquez, (b, c and d) Pedro Taucce, (f) Francisco Severo Neto and (g and h) Iuri Dias.

## Additional Information and Declarations

### DNA Deposition

The following information was supplied regarding the deposition of DNA sequences: Sequences of the two paratypes of *Phasmahyla lisbella* sp. nov. have been deposited at GenBank (accession numbers: MG954000, MG954001). Raw sequence data can be found in Document S3.

### Data Availability

The following information was supplied regarding data availability: The specimens used in this paper are housed at Coleção Zoológica da Universidade Federal de Mato Grosso do Sul: ZUFMS-AMP 08803 (holotype), ZUFMS-AMP 8804-06, ZUFMS-AMP 8808-09, 8811; and Museu de Zoologia João Moojen, Univerisdade Federal de Viçosa: MZUFV 18685, MZUFV 18686.

### New Species Registration

The following information was supplied regarding the registration of a newly described species: Publication LSID: urn:lsid:zoobank.org:pub:DAD55134-8943-4189-AF4A-7004FA5FCD51; Taxon LSID: Phasmahyla lisbella: urn:lsid:zoobank.org:act:48E90F44-394E-4C53-BEDA-0230585524A8.
