# Supplementary material for: A new species of spotted leaf frog, genus Phasmahyla (Amphibia, Phyllomedusidae) from Southeast Brazil
Source: PeerJ. 2018 May 30;6:e4900. doi: 10.7717/peerj.4900 (PMC5984584; doi:10.7717/peerj.4900)
Supplement: Supplemental Information 1 [file peerj-06-4900-s001.docx]

Online Supplementary data – A new species of spotted leaf frog genus *Phasmahyla*

**Supplementary document 1.** List of sequences from GenBank used in this study (16S rRNA).

| GenBank accession number | Species | Reference |
| --- | --- | --- |
| KF955305.1 | *Boana alfaroi* | Caminer & Ron, 2013 |
| AY843715 | *Phasmahyla cochranae* | Faivovich et al., 2004 |
| KU495415 | *Phasmahyla cochranae* | Lyra et al., 2016 |
| KU495416 | *Phasmahyla cochranae* | Lyra et al., 2016 |
| KU495417 | *Phasmahyla cochranae* | Lyra et al., 2016 |
| KU517258 | *Phasmahyla cruzi* | Vences et al., 2016 |
| KU517344.1 | *Phasmahyla cruzi* | Vences et al., 2016 |
| KU517345 | *Phasmahyla cruzi* | Vences et al., 2016 |
| KU517346.1 | *Phasmahyla cruzi* | Vences et al., 2016 |
| KU517350 | *Phasmahyla cruzi* | Vences et al., 2016 |
| KU517351.1 | *Phasmahyla cruzi* | Vences et al., 2016 |
| KU517352.1 | *Phasmahyla cruzi* | Vences et al., 2016 |
| KU517353.1 | *Phasmahyla cruzi* | Vences et al., 2016 |
| KU517354.1 | *Phasmahyla cruzi* | Vences et al., 2016 |
| KU517355.1 | *Phasmahyla cruzi* | Vences et al., 2016 |
| KU517356 | *Phasmahyla cruzi* | Vences et al., 2016 |
| KU517357 | *Phasmahyla cruzi* | Vences et al., 2016 |
| GQ366231 | *Phasmahyla exilis* | Faivovich et al., 2009 |
| AY843716 | *Phasmahyla guttata* | Faivovich et al., 2004 |
| GQ366232.1 | *Phasmahyla guttata* | Faivovich et al., 2009 |
| GQ366233 | *Phasmahyla jandaia* | Faivovich et al., 2009 |
| MG954000 | *Phasmahyla lisbella* sp. nov. | This study |
| MG954001 | *Phasmahyla lisbella* sp. nov. | This study |
| KM387517 | *Phasmahyla spectabilis* | Barth et al., 2014 |
| JF789937 | *Phyllomedusa camba* | Bloch et al., 2011 |
| KU495425 | *Phyllomedusa distincta* | Lyra et al., 2016 |
| GQ366269 | *Phyllomedusa megacephala* | Faivovich et al., 2009 |
| GQ366236 | *Phyllomedusa rohdei* | Faivovich et al., 2009 |
