# Supplementary material for: A new species of spotted leaf frog, genus Phasmahyla (Amphibia, Phyllomedusidae) from Southeast Brazil
Source: PeerJ. 2018 May 30;6:e4900. doi: 10.7717/peerj.4900 (PMC5984584; doi:10.7717/peerj.4900)
Supplement: Supplemental Information 3 [file peerj-06-4900-s003.docx]

>Phasmahyla_lisbella_MG954000
NNNNGNNTCNNNNNNNCNNGCTGCCCAGTGACTTTGTTTAACGGCCGCGGTATCCTAACCGTGCGAAGGTAGTGCAATCACTTGTTCTTTAAATGAGGACTAGTATGAACGGCATCACGAAGGTTACACTGTCTCCTTTCTCTAATCAGTGAAATTGATTTTCCCGTGAAGAAGCGGGAATAAATATATAAGACGAGAAGACCCTATGGAGCTTTAAACTATTAACAATTATTTTTTCTTAACAAACCTTAAGGCTACAAAATTAATTTAAATATTCTGTTTACCAGTTTTCGGTTGGGGTGACCACGGAGTAAAACTTATCCTCCACGATGAATTAGAGCCCGTCTCTAAGCAAAAAACTACAATTTTAAGCATCAAAAAATTGACTCTATTGACCCAATATTTGATCAACGAACCAAGTTACCCTATGGATAACAGCGCAATCCATCTCAAGAGCTCATATCGACAGATGGGTTTACGACCTCGATGTTGGATCAGGATGTCCAAGTGGTGCAGCCGCTACTAAAGGTTCGTTTGTTCAACGATTAAAATCCTACGTGATCTGATTCCCAGAACCGGA

>Phasmahyla_lisbella_MG954001
GNACNNNCTCNATNNNNGGNATGCCCAGTGACTTTGTTTAACGGCCGCGGTATCCTAACCGTGCGAAGGTAGCGTAATCACTTGTTCTTTAAATGAGGACTAGTATGAACGGCATCACGAAGGTTACACTGTCTCCTTTCTCTAATCAGTGAAATTGATTTTCCCGTGAAGAAGCGGGAATAAATATATAAGACGAGAAGACCCTATGGAGCTTTAAACTATTAACAATTATTTTTTCTTAACAAACCTTAAGGCTACAAAATTAATTTAAATATTCTGTTTACCAGTTTTCGGTTGGGGTGACCACGGAGTAAAACTTATCCTCCACGATGAATTAGAGCCCGTCTCTAAGCAAAAAACTACAATTTTAAGCATCAAAAAATTGACTCTATTGACCCAATATTTGATCAACGAACCAAGTTACCCTAGGGATAACAGCGCAATCCATCTCAAGAGCTCATATCGACAGATGGGTTTACGACCTCGATGTTGGATCAGGATGTCCAAGTGGTGCAGCCGCTACTAAAGGTTCGTTTGTTCAACGATTAAAATCCTACGTGATCTGAGTTCAGACCGG
