# Supplementary figures and images for: A new species of spotted leaf frog, genus Phasmahyla (Amphibia, Phyllomedusidae) from Southeast Brazil
Source: PeerJ. 2018 May 30;6:e4900. doi: 10.7717/peerj.4900 (PMC5984584; doi:10.7717/peerj.4900)

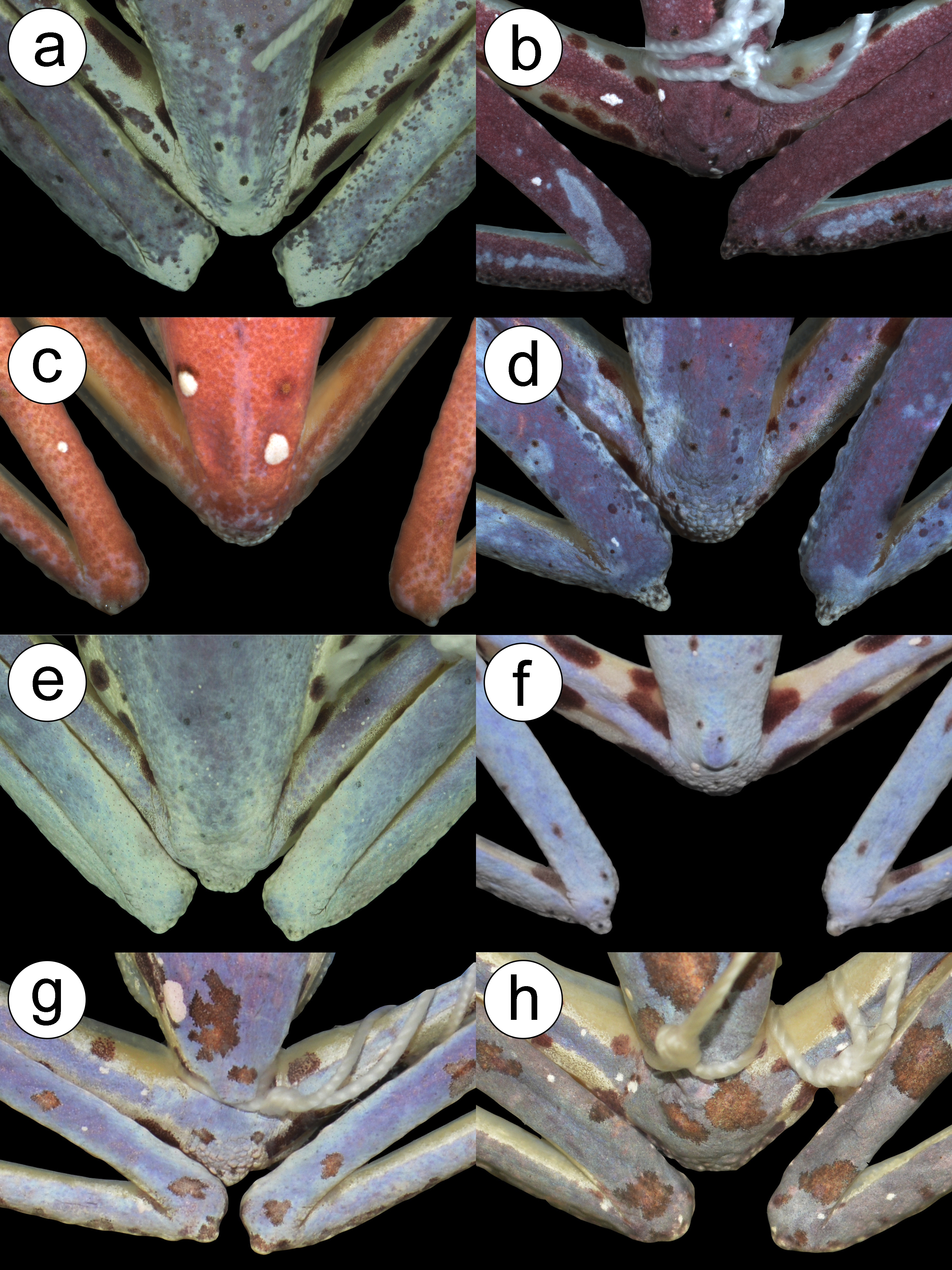

Supplement: Supplemental Information 4 — a) P. cochranae (UFMG-AMP 1564); b) P. cruzi (CFBH 39151); c) P. exilis (CFBH 4022); d) P. guttata (CFBH 05704); e) P. jandaia (UFMG-AMP 19641); f) P. lisbella sp. nov. (ZUFMS-AMP 08803, holotype); g) P. spectabilis (MZUESC 16616); h) P. timbo (MZUESC 16609, topotype). Image credit/source: (a and e) Sofia Velasquez, (b, c and d) Pedro Taucce, (f) Francisco Severo Neto and (g and h) Iuri Dias. [file peerj-06-4900-s004.jpg]
